# Scientific American

**Published:** 1884-02

**Authors:** 


### SCIENTIFIC AMERICAN.

This is a weekly journal of practical information, art, science, mechanics, chemistry, and manufactures, published in New York by Munn & Co. It is an invaluable journal to all who have any interest in the subjects to which it pertains. All classes of readers will find in the *Scientific American* a popular resume of the best scientific information of the day. Each number contains sixteen pages of useful information, with a large number of original engravings of new inventions and discoveries pertaining to engineering, steam machinery, new inventions in manufactures, chemistry, electricity, telegraphy, photography, architecture, agriculture, horticulture, natural history, etc. All these subjects are treated in an attractive and understandable form; technicalities and abstruse terms are avoided as far as possible.

Any one who desires to keep abreast with the progress of the times must have this or some similar journal, and so far as meeting the want in these matters, the *Scientific American* has no superior and probably not an equal in the world. It affords a constant supply of instructive reading, and is promotive of knowledge and progress in every community where it circulates.

The *Scientific American* should be in the hands of every intelligent person in the country, who has, or ought to have, an interest in the subjects of which it treats. Let every reader of the REGISTER send to Munn & Co., 261 Broadway, New York, for the journal, and he will find that not the half is here told.

